# Correction: Celiksoy et al. Evaluation of the In Vitro Oral Wound Healing Effects of Pomegranate (*Punica granatum*) Rind Extract and Punicalagin, in Combination with Zn (II). *Biomolecules* 2020, *10*, 1234

**DOI:** 10.3390/biom16020287

**Published:** 2026-02-11

**Authors:** Vildan Celiksoy, Rachael L. Moses, Alastair J. Sloan, Ryan Moseley, Charles M. Heard

**Affiliations:** 1 School of Pharmacy and Pharmaceutical Sciences, Cardiff University, Cardiff CF10 3NB, UK; celiksoyv@cardiff.ac.uk; 2 Oral and Biomedical Sciences, School of Dentistry, Cardiff University, Cardiff CF14 4XY, UK; mosesr@cardiff.ac.uk; 3 Melbourne Dental School, Faculty of Medicine, Dentistry and Health Sciences, University of Melbourne, Melbourne, VIC 3010, Australia; alastair.sloan@unimelb.edu.au

In the original publication [1], there was a mistake in Figure 4 as published. One of the images originally included in Figure 4 for the PRE 1 µg/mL and Zn (II) 0.1 mM experimental condition was included in error, as it is a duplicate of another image correctly included in this Figure for the PRE 0.1 µg/mL and Zn (II) 0.1 mM experimental condition. The corrected Figure 4 appears below. 

Affiliation 3 was also updated to the correct format, “Melbourne Dental School, Faculty of Medicine, Dentistry and Health Sciences, University of Melbourne, Melbourne, VIC 3010, Australia”.

The authors state that the scientific conclusions are unaffected. This correction was approved by the Academic Editor. The original publication has also been updated.

## Figures and Tables

**Figure 4:**
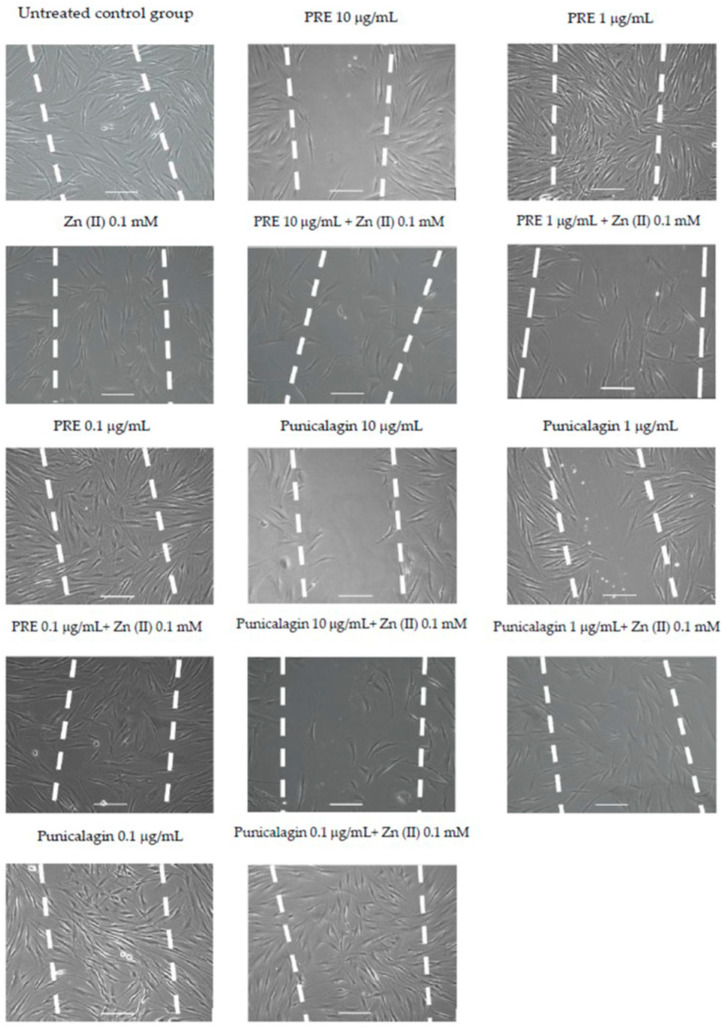
Representative time-lapse microscopy images of gingival fibroblast migration and wound repopulation at 48 h, following treatment with PRE and punicalagin (0.1–10 μg/mL) alone and with 0.1 mM Zn (II), White dashed lines show original scratch wounds at 0 h. Scale bar = 100 μm.

## References

[1] Celiksoy V., Moses R.L., Sloan A.J., Moseley R., Heard C.M. (2020). Evaluation of the In Vitro Oral Wound Healing Effects of Pomegranate (*Punica granatum*) Rind Extract and Punicalagin, in Combination with Zn (II). Biomolecules.

